# Translating a walking intervention for health professional delivery within primary care: A mixed‐methods treatment fidelity assessment

**DOI:** 10.1111/bjhp.12392

**Published:** 2019-11-19

**Authors:** Stefanie L. Williams, Jennifer McSharry, Claire Taylor, Jeremy Dale, Susan Michie, David P. French

**Affiliations:** ^1^ Centre for Intelligent Healthcare Coventry University Coventry UK; ^2^ Health Behaviour Change Research Group School of Psychology NUI Galway Galway Ireland; ^3^ Public Health Warwickshire Warwickshire County Council Warwick UK; ^4^ Warwick Medical School University of Warwick Coventry UK; ^5^ Department of Clinical, Educational and Health Psychology University College London London UK; ^6^ Manchester Centre for Health Psychology School of Health Sciences University of Manchester UK

**Keywords:** general practice, implementation, intervention, intervention fidelity, physical activity, primary care, treatment fidelity, walking

## Abstract

**Objectives:**

Existing fidelity studies of physical activity interventions are limited in methodological quality and rigour, particularly those delivered by health care providers in clinical settings. The present study aimed to enhance and assess the fidelity of a walking intervention delivered by health care providers within general practice in line with the NIH Behavior Change Consortium treatment fidelity framework.

**Design:**

Two practice nurses and six health care assistants delivered a theory‐based walking intervention to 63 patients in their own practices. A cross‐sectional mixed‐methods study assessed fidelity related to treatment delivery and treatment receipt, from the perspectives of health care providers and patients.

**Methods:**

All providers received training and demonstrated delivery competence prior to the trial. Delivery of intervention content was coded from audio‐recordings using a standardized checklist. Qualitative interviews with 12 patients were conducted to assess patient perspectives of treatment receipt and analysed using framework analysis.

**Results:**

Overall, 78% of intervention components were delivered as per the protocol (range 36–91%), with greater fidelity for components requiring active engagement from patients (e.g., completion of worksheets). The qualitative data highlighted differences in patients’ comprehension of specific intervention components. Understanding of, and engagement with, motivational components aimed at improving self‐efficacy was poorer than for volitional planning components.

**Conclusions:**

High levels of fidelity of delivery were demonstrated. However, patient‐, provider‐, and component‐level factors impacted on treatment delivery and receipt. We recommend that methods for the enhancement and assessment of treatment fidelity are consistently implemented to enhance the rigour of physical activity intervention research.

**Statement of contribution**

***What is already known on this subject?***

- Physical activity interventions delivered within primary care by health professionals have so far demonstrated limited impact on behaviour change initiation and maintenance.
- Treatment fidelity enhancement and assessment strategies can support the successful translation of behaviour change interventions into real‐life settings.
- Few studies have examined treatment fidelity within the context of physical activity interventions, particularly within clinical settings, and existing fidelity studies are limited by methodological quality and rigour.

***What does this study add?***

- High levels of fidelity were found for a physical activity intervention delivered in primary care.
- Patient‐, provider‐, and component‐level factors may impact on treatment delivery and receipt.
- The implementation of best practice fidelity recommendations can support near‐optimal fidelity.

## Background

Physical inactivity is the fourth leading mortality risk factor worldwide (World Health Organization, 2017) and is associated with increased risk of chronic disease, and premature mortality (Lee *et al*., 2012). Therefore, the World Health Organization (2010) and the UK Departments of Health (2011) recommend that adults achieve 150 min of moderate‐intensity, or 75 min of vigorous‐intensity, aerobic physical activity (PA) per week to achieve health benefits. Despite this, in England only 66% of men and 58% of women report meeting these recommendations (Fuller, Mindell, & Prior, 2017).

Walking is a low‐cost PA which is accessible and convenient to most populations (Morris & Hardman, 1997). Regular brisk walking is associated with decreased body weight and resting diastolic blood pressure in previously sedentary adults (Murphy, Nevill, Murtagh, & Holder, 2007; Ogilvie *et al*., 2007). Thus, promoting brisk walking is a promising target for public health interventions.

An advantage of primary care as a setting for physical activity promotion is that a significant proportion of the general population have regular contact with primary care providers (Williams, 2011). As such, adults who are not meeting UK physical activity recommendations should be identified and offered brief advice within general practice by primary care practitioners (NICE, 2013). The current study investigates treatment fidelity of a walking intervention delivered in primary care by health care providers. The walking intervention was based on a similar intervention used in volunteer populations which has been shown to yield large increases in objectively measured walking behaviour outside of primary care (Darker, French, Eves, & Sniehotta, 2010; French, Stevenson, & Michie, 2012).

Despite the potential impact of PA interventions in primary care, modest effects of interventions were found in a recent review of reviews of PA promotion trials within this setting (Sanchez, Bully, Martinez, & Grandes, 2015). Furthermore, PA interventions delivered within primary care by health professionals have been shown to demonstrate less effectiveness in achieving behaviour change maintenance compared to those delivered within community settings, and by researchers (Murray *et al*., 2017). Therefore, whilst primary care presents an opportunity for health behaviour change, there is a lack of compelling evidence on effective interventions delivered within this setting.

The challenge of translating behaviour change interventions from a controlled research environment to real‐life setting may contribute to the lack of effective PA interventions delivered in primary care (Glasgow, Lichtenstein, & Marcus, 2003). The complexity of organizational and contextual issues inherent within primary care can impact upon the ability of practitioners to effectively deliver health behaviour change interventions as intended (Taylor, Shaw, Dale, & French, 2011). Thus, non‐significant results from studies in this setting may reflect interventions not being delivered as intended, rather than lack of intervention efficacy.

Treatment fidelity refers to ‘the methodological strategies used to monitor and enhance the reliability and validity of behavioural interventions’ and considers the extent to which such interventions are delivered in line with the underlying theory and intervention protocols (Bellg *et al*., 2004). In 2004, the National Institute of Health Behavior Change Consortium (NIH‐BCC) developed a comprehensive treatment fidelity framework incorporating five areas: design of study, training providers, delivery of treatment, and receipt of treatment and enactment of treatment skills (Bellg *et al*., 2004). For example, enhancing fidelity related to study design necessitates defining and specifying intervention content and the dose of delivery, the latter of which should be monitored over time (Borelli, 2011). Assessing fidelity of delivery can also help to determine information regarding dose effects (Resnick *et al*., 2005); for example, participants may experience the intended behavioural outcomes in less intervention sessions than originally intended, thus enhancing cost‐effectiveness and efficiency (Bellg *et al*., 2004; Borelli, 2011; Moncher & Prinz, 1991).

It has been recommended that the assessment of treatment fidelity should become an integral part of behaviour change intervention development and implementation (Bellg *et al*., 2004; Borelli, 2011; Resnick *et al*., 2005). The extent to which this has been established is, however, varied. The assessment of treatment fidelity of smoking cessation interventions delivered within clinical practice has been undertaken using reliable and rigorous methods. Lorencatto, West, Christopherson, and Michie (2013) assessed the treatment fidelity of face‐to‐face English NHS Stop Smoking Services by coding the delivery of behavioural support session content using a standardized smoking cessation behaviour change technique (BCT) taxonomy (Michie, Hyder, Walia, & West, 2011). Fidelity levels were 66% across two services, with substantial variation found across session type, practitioner, and BCT type. However, substantially lower mean fidelity levels (41%) were found within a telephone smoking cessation Quitline (Lorencatto, West, Bruguera, & Michie, 2014). Further, a recent review of fidelity monitoring within seven US clinical trials of smoking cessation interventions (Duffy *et al*., 2015) identified that a variety of objective, self‐report, and automated fidelity measures had been implemented, encompassing multiple fidelity domains. The review found that provider training was implemented across all studies examined, four studies used checklists to monitor delivery, five studies assessed patient receipt, and all assessed treatment enactment. Thus, the successful implementation of treatment fidelity assessments of smoking cessation interventions delivered by health care providers within clinical settings has been demonstrated.

By contrast, treatment fidelity remains under‐researched in the PA literature (Lambert *et al*., 2017; Quested, Ntoumanis, Thøgersen‐Ntoumani, Hagger, & Hancox, 2006). When fidelity is investigated in studies of PA interventions, significant heterogeneity in methodological rigour and quality has been identified. A 2017 systematic review of treatment fidelity in PA behaviour change trials found only one study ensured providers were trained to a well‐defined *a priori* performance criterion, few studies used objective data collection methods to assess delivery; and the quality, expertise, and reliability of fidelity raters were rarely examined (Lambert *et al*., 2017). Likewise, assessment of treatment receipt, that is, participant's understanding of the intervention content, was rarely investigated.

Further, a recent systematic review investigating the psychometric and implementation quality of fidelity measures found few studies that investigated treatment fidelity from the perspective of both the provider (treatment delivery) and the participants (receipt and enactment) (Walton, Spector, Tombor, & Michie, 2017). Of the studies that did explore both perspectives, few investigated treatment fidelity in a clinical setting and most recruited providers specifically employed and trained to deliver the intervention (Walton *et al*., 2017). Thus, treatment fidelity issues which may arise when primary care providers deliver interventions to their usual patients alongside their routine work have not previously been explored in depth.

In the current study, the NIH‐BCC framework (Bellg *et al*., 2004) was used to both enhance and assess treatment fidelity of a walking intervention delivered by health care providers within primary care. As such, best practice recommendations encompassing the five domains present within the NIH‐BCC framework (Bellg *et al*., 2004), that is, treatment design, training, delivery, receipt and enactment, were implemented. The current paper details the implementation of the first four of these recommendations within a pilot trial of the intervention, with a specific focus on the assessment of fidelity related to treatment delivery and treatment receipt. More detailed information on treatment design, receipt, and enactment can be found elsewhere (French *et al*., 2011; Williams, Michie, Dale, Stallard, & French, 2015).

The present study aimed to assess treatment delivery by investigating health care providers’ adherence to a walking intervention protocol in terms of content and dose and to assess treatment receipt by exploring patients’ understanding and experiences of the intervention. Specifically, the study aimed to (1) enhance provider competency to deliver the walking intervention through health care provider training and competency assessments, (2) assess the extent to which health care providers adhere to the specified components defined in the walking intervention protocol (intervention content), (3) assess the time spent delivering individual intervention components and the overall intervention (intervention dose), (4) assess differences in fidelity of delivery of the walking intervention across providers, and (5) explore the extent to which patients understood the intervention content (patient receipt).

## Methods

### Design

A cross‐sectional design using observational methods investigated the extent to which Practice Nurses (PNs) and Health Care Assistants (HCAs) adhered to the pre‐specified intervention protocol, and dose of delivery (treatment delivery). A cross‐sectional design using qualitative methods investigated treatment receipt in a subsample of patients.

### Participants

Participants were recruited from GP practices within Coventry and Warwickshire Primary Care Trusts between 2009 and 2010.

Seven GP practices participated, from which eight PNs and HCAs were recruited to deliver the intervention to up to 15 of their own eligible patients. Practices were encouraged to provide protected time for PNs and HCAs to deliver the intervention, and excess treatment costs were provided to reimburse staff costs.

Patients at these practices were eligible for inclusion in the study if they were (1) aged between 16 and 65 years, (2) had one or more chronic conditions for which increasing PA would have a positive effect on health status, and (3) were inactive in terms of not meeting government physical activity guidelines. Patients were identified from registers at GP practices at which the study was based and were sent brief letters inviting them to participate if they met the inclusion criteria. Further information on recruitment methods, and details of the cluster randomized controlled trial of the walking intervention can be found elsewhere (French *et al*., 2011; Williams *et al*., 2015).

### Procedure

#### Enhancement of treatment fidelity: Treatment delivery

The walking intervention, based on the Theory of Planned Behaviour and Social Cognitive Theory (Ajzen, 1991; Bandura, 1997; Sniehotta, Schwarzer, Scholz, & Schüz, 2005), consisted of components designed to increase walking self‐efficacy (confidence in ability to perform the behaviour) and to help people translate their ‘good’ intentions into behaviour change by making plans. The intervention comprised two face‐to‐face sessions, 1 week apart, with a telephone or face‐to‐face follow‐up 2 weeks after the second session. In accordance with NIH‐BCC recommendations (Bellg *et al*., 2004), an apriori specification of dose of delivery (i.e., duration) was determined. The suggested duration (dose) for delivery of intervention content was 25 min for Session One and 26 min for Session Two. The duration required to deliver each session with optimum fidelity was assessed during earlier development work. Optimum delivery of the walking intervention by a Research Nurse was observed for ten patients and recorded to the nearest minute, thus forming the basis for decisions regarding recommended dose.

Session One included motivational components to increase the patients’ walking self‐efficacy, and volitional components to facilitate realistic goal setting and to translate intentions into practice. Session One comprised six main sections (Introduction, Assessment of Average Daily Walking, What Makes it Easier to Walk, Walking Experiences, Goal Setting, and Action Planning/Conclusions). Between one and six distinct intervention components were included in each section. For instance, within the ‘Goal Setting’ section (section five), providers were expected to deliver two distinct intervention components, that is, patient chooses a goal to increase walking by 10 or 20 min/day (component 5.1), and patient chooses a goal (component 5.2). A total of 14 intervention components were included in Session One.

Session Two included a review of progress with positive feedback for effort and achievement, and revision of goals and action plans. Session Two also included supportive planning, whereby the participant was encouraged to identify what factors support increased walking and make plans to bring about those factors. Session Two consisted of five main sections (Introduction, Review of Behaviour Change, Goal Re‐evaluation/setting, Supportive Planning, Action Planning/Conclusions) and 15 distinct intervention components. The follow‐up session consisted of an abbreviated version of the components delivered in Session Two.

See Table 1 and TIDieR checklist (Appendix S1) for a full description of intervention components, described in terms of both the intervention protocol definition and Behaviour Change Technique Taxonomy V1 classifications (Michie *et al*., 2013). Detailed information on the development of the intervention can be found elsewhere (French *et al*., 2011; Williams *et al*., 2015).

**Table 1:** Specified sections and intervention components for Sessions One and Two of the walking intervention

Section	Intervention components		BCT taxonomy (V1) classification*
Session one			
1. Introduction	1.1	Outline of session provided	5.1. Information about health consequences 5.4 Information about emotional consequences
2. Assessment of Average Daily Walking	2.1	Average daily walking communicated to the patient	2.2 Feedback on behaviour
3. What Makes it Easier to Walk (WMIETW)	3.1	Patient asked to complete WMIETW worksheet	1.2 Problem solving
	3.2	Patient asked to elaborate on WMIETW worksheet responses	
4. Walking Experiences (WE)	4.1	Patient asked to complete WE worksheet	1.2 Problem solving 5.4 Monitoring of emotional consequences 15.3 Focus on past success
	4.2	Patient asked to elaborate on WE worksheet responses	
5. Goal Setting	5.1	Patient offered a goal	1.1 Goal setting (behaviour) 1.9 Commitment 8.1 Prompt practice/rehearsal
	5.2	Patient chooses goal	
6. Action Planning (AP)/Conclusions	6.1	Patient asked to complete action plan	1.4 Action planning 2.3 Self‐monitoring of behaviour 3.2 Social support (practical) 7.1 Prompts/cues 8.1 Prompt practice/rehearsal 12.1 Restructuring the physical environment 12.2 Restructuring the social environment
	6.2	Patient asked to elaborate on action plan	
	6.3	Patient asked to complete walking diary	
	6.4	Patient asked to summarize the session	
	6.5	Patient asked to summarize their plans for walking	
	6.6	Positive end to the session	
Session two			
7. Introduction	7.1	Outline of session provided	
8. Review of behaviour change	8.1	Patient asked to describe walks completed in intervening week	1.2 Problem solving 2.2 Feedback on behaviour 3.1 Social support (unspecified) 8.1 Prompt practice/rehearsal 10.4 Social reward
	8.2	Patient informed of average daily walking	
	8.3	Patient praised for their efforts	
	8.4	Patient asked to elaborate on what helped them to walk more	
9. Goal re‐evaluation/setting	9.1	Patient offered a goal	1.1 Goal setting (behaviour) 1.2 Problem solving 1.5 Review behaviour goal(s) 1.6 Discrepancies between current behaviour and goal 8.1 Prompt practice/rehearsal 8.7 Graded tasks
	9.2	Patient chooses a goal	
10. Supportive Planning	10.1	Patient asked to complete supportive plan worksheet	1.2 Problem solving 1.9 Commitment 3.1 Social support (unspecified) 3.2 Social support (practical) 8.1 Prompt practice/rehearsal 12.1 Restructuring the physical environment 12.2 Restructuring the social environment 15.2 Mental rehearsal of successful performance
	10.2	Patient asked to elaborate supportive plan worksheet	
11. Action Planning/Conclusions	11.1	Patient asked to complete action plan	1.4 Action planning 2.3 Self‐monitoring of behaviour 3.1 Social support (unspecified) 3.2 Social support (practical) 7.1 Prompts/cues 8.1 Prompt practice/rehearsal 12.1 Restructuring the physical environment 12.2 Restructuring the social environment
	11.2	Patient asked to elaborate on action plan	
	11.3	Patient asked to complete walking diary	
	11.4	Patient asked to summarize session	
	11.5	Patient asked to summarize plans for walking	
	11.6	Positive end to the session	

* Behaviour Change Technique Taxonomy (V1) (Michie *et al*., 2013).

#### Enhancement of treatment fidelity: Provider training and competency assessments

Each provider received training to facilitate effective delivery of the walking intervention. This consisted of two half‐day training sessions, 1 week apart, delivered by members of the research team. Each training session consisted of formal presentations, video demonstration of delivery of the intervention, participative learning, and practice sessions.

Providers were subsequently observed delivering the session content to one colleague or patient within their own practice prior to entering the pilot trial. Delivery was assessed by one of the authors using a checklist of essential intervention components, as specified in the intervention protocol. Providers were given immediate verbal feedback, followed up with written feedback, with the aim of enhancing the skills and confidence of the provider.

For *intervention sections*, a score of 1 was given where a section was delivered in the correct order. For *intervention components*, a score of 1 was given when a component was delivered as specified in the protocol. Providers were required to achieve 15 or more marks out of a maximum of 20, reflecting correct delivery of at least 15 intervention sections and components, to be certified as competent to deliver the intervention.

#### Assessment of treatment fidelity related to delivery

Each provider was given a digital audio‐recorder with instructions to record all intervention sessions. Research team visits and the offer of a £25 gift voucher were used to encourage recording of all intervention sessions, text message and email prompts were also used.

#### Assessment of treatment fidelity related to receipt

A subsample of patients who received the walking intervention was invited to participate in an interview to explore their understanding and experiences. Patients from each of the included providers were invited for interview, and a purposive sampling strategy was used to maximize the variability of patients interviewed (Ritchie, Lewis, & Elam, 2003) in terms of gender, age, and presence of health conditions.

Face‐to‐face interviews were undertaken within 1 week of patients’ receipt of Session Two. Interviews were conducted either at the patients’ home or at the GP practice. All interviews were audio‐recorded and transcribed verbatim.

A semi‐structured interview schedule was used to explore patients’ experiences of participating in the intervention, including their (1) decision to participate in the research, (2) expectations of the intervention, (3) understanding and comprehension of each part of the intervention including specific components, (4) perceptions of the intervention resources, and (5) views on the role of the provider in delivering the intervention. Probes and prompts were used to further explore patients’ responses. The interviews were conducted by a single researcher (CT). The interview schedule used in the present study is available (see Appendix S2).

### Analysis

#### Analysis of treatment fidelity related to delivery

In total, 50% of the delivered intervention sessions were coded across all providers and evenly distributed across the period of delivery, in accordance with the NIH‐BCC framework (Bellg *et al*., 2004). A coding frame developed specifically for the present study was used (see Appendix S3). If up to five intervention sessions were recorded by a provider, all were included. If more than five sessions were recorded, then five were included. The subsample for coding was obtained by using the first, middle, and last delivered and recorded session, and the session mid‐way between the first and middle, and the middle and last session.

Coding was conducted on half (*n* = 62, 51%) of the 122 intervention sessions delivered across all providers, evenly distributed across the period of delivery. In total, *n* = 32 out of a total of *n* = 63 (51%) deliveries of Session One, and *n* = 30 out of a total of *n* = 59 (54%) deliveries of Session Two of the intervention were coded. Coding was undertaken by one researcher (CT) who was independent of the core research team for the pilot trial. 25% of the sessions were double‐coded by a second coder who was independent from the research team and chance‐corrected Cohen's Kappa coefficient was used to measure agreement.

The delivery of intervention sections (*n* = 11), and their respective intervention components (*n* = 29), was coded across both intervention sessions. Coding of content was undertaken in accordance with the criteria applied during assessment of provider competence stated above. The length of time spent delivering sessions and intervention components was coded to assess dose of delivery. The section ‘Action Planning/Conclusions’ was separated into two distinct sections for the purposes of assessing dose of delivery. The overall length of the session was calculated from the formal welcome to the session by the provider to the time when the intervention delivery was deemed to be complete, usually when the provider thanked the patient for attending and the patient was heard leaving the room.

#### Analysis of treatment fidelity related to receipt

A framework analysis was conducted (Ritchie & Spencer, 1994) to examine patient receipt of the intervention. A flexible thematic framework was developed by the research team to capture important elements of intervention receipt and was subsequently further refined in an iterative process to encompass other aspects of patient interaction with the intervention, that is, expectations, acceptability, and engagement. The analysis followed the stages of familiarization, identifying a thematic framework, indexing, charting and mapping and interpretation (Ritchie & Spencer, 1994). The analysis was conducted primarily by a single researcher with ongoing discussion with members of the research team regarding themes as the analysis progressed to ensure credibility of findings, this focused on the nature of the framework, and how the transcribed material fit within the framework. The framework used in the present study is available (see Appendix S4).

## Results

### Participant characteristics

Eight providers, comprising two PNs and six HCAs delivered the intervention. One PN and one HCA were from the same practice. All were female, White British, and were aged between 24 and 57 years old (*M* = 43 years, *SD* = 10.2). All had been working in their present role for at least 1 year.

A total of 63 patients received the intervention during the pilot trial, with between 3 and 14 patients receiving the intervention from each provider. Patients were aged between 30 and 75 years (mean = 56 years, *SD* = 8.5). Three quarters of the sample (74%) were female and 87% of patients were British White. Twelve patients (50% female) were interviewed, nine at home, and three at their general practice. Of those interviewed, four patients were aged between 16 and 54 years and eight were aged 55 years or over, 92% of patients interviewed were British White. Six of the patients had long‐term conditions such as diabetes, asthma, or heart disease, and six patients had hypertension, high cholesterol levels, were overweight or obese.

### Treatment fidelity related to delivery

Inter‐rater reliability for coding intervention sections and components was good across both intervention sessions with all kappas between 0.69 and 0.78, indicating substantial agreement across raters (McHugh, 2012).

Overall 78% of intervention components were delivered as specified in the protocol. The percentage of sessions delivered as per the protocol was 80% for Session One (of 32 Session Ones coded) and 76% for Session Two (of 30 Session Twos coded, see Tables 2 and 3). Intervention sections were delivered in the right order in over 75% of Session One (range 53–100%) and 65% of Session Two deliveries (range 57–70%). Intervention components were delivered according to protocol in 82% of Session One (range 36–100%) and 79% of Session Two (range 40–100%) deliveries.

**Table 2:** Overall fidelity of delivery of sections and intervention components coded for Session One

	Name of section/intervention component	% Sessions delivered
1	Introduction	84
1.1	Overview of Session	84
2.	Assessment of average daily walking	53
2.1	Average daily walking communicated to the patient	94
3	What Makes It Easier to Walk? (WMIETW)	53
3.1	Patient asked to complete WMIETW worksheet	100
3.2	Patient asked to elaborate on WMIETW worksheet responses	75
4	Walking Experiences (WE)	100
4.1	Patient asked to complete WE worksheet	100
4.2	Patient asked to elaborate on WE worksheet responses	94
5.	Goal Setting	69
5.1	Patient offered a goal	78
5.2	Patient chooses a goal	84
6	Action Planning (AP)/Conclusions	94
6.1	Patient asked to complete AP	100
6.2	Patient asked to elaborate on AP	91
6.3	Patient asked to complete walking diary	100
6.4	Patient asked to summarize the session	36
6.5	Patient asked to summarize their plans for walking	41
6.6	Positive end to the session	72
Total		80

**Table 3:** Overall fidelity of delivery of sections and intervention components coded for Session Two

	Name of section/intervention component	% Sessions delivered
7	Introduction	70
7.1	Overview of Session	70
8.	Review of Behaviour Change	70
8.1	Patient asked to describe walks	87
8.2	Average daily walking communicated	90
8.3	Positive feedback	93
8.4	What helped patient to walk more	87
9	Goal re‐evaluation/setting	57
9.1	Patient offered a goal	73
9.2	Patient chooses a goal	93
10	Supportive Planning (SP)	60
10.1	Patient asked to complete SP worksheet	100
10.2	Patient asked to elaborate on SP worksheet	87
11	Action Planning (AP)/Conclusions	70
11.1	Patient asked to complete AP	93
11.2	Patient asked to elaborate on AP	83
11.3	Patient asked to complete walking diary	70
11.4	Patient asked to summarize the session	43
11.5	Patient asked to summarize their plans for walking	40
11.6	Positive end to the session	73
Total		76

### Intervention content

There was variation in the extent to which intervention sections were delivered in the specified order. During Session One, ‘Assessment of Average Daily Walking’ (section two) and ‘What Makes it Easier to Walk?’ (section three) were delivered in the specified place in just over half of all sessions (17/32). By contrast, ‘Walking Experiences’ (section four) was delivered in the specified place in 100% of coded sessions, and ‘Action Plan’ was delivered in the correct place during 94% of Session One deliveries (section six). In Session Two, the ‘Goal re‐evaluation/setting’ section (section nine) was delivered in the specified place in 57% (17/30) of the sessions. ‘Supportive Planning’ (section ten) was delivered in the specified place in 60% (18/30) of sessions, and both ‘Action Planning/Conclusions’ (section eleven) and ‘Review of behaviour change’ (section eight) were delivered in the correct place in 70% (21/30) of Session Two deliveries.

Fidelity varied across intervention components. Asking patients to complete the worksheets ‘What Makes It Easier to Walk’ (component 3.1) in Session One and ‘Supportive Plan’ (component 10.1) in Session Two was delivered with fidelity in 100% of coded sessions. Asking patients to elaborate on these worksheets was delivered with less fidelity in both Session One and Session Two (components 3.2 and 10.2). Asking patients to complete ‘Action Planning’ was completed in 100% of coded Session One (Component 6.1) and 93% of Session Two recordings (Component 11.1).

By contrast, the components ‘Summary of Session’ (components 6.4 and 11.4) and ‘Summary of Plans’ (components 6.5 and 11.5) were delivered with least fidelity in both sessions, both were delivered in fewer than 50% of sessions. In every Session One coded, patients were asked to complete their ‘Walking Diary’ (component 6.3); in Session Two, patients were asked to complete the ‘Walking Diary’ (component 11.3) in 22 of 30 (73%) sessions.

### Intervention dose

In most cases, the overall intervention was delivered in less time than recommended to providers (see Table 4). For Session One, mean delivery time was 19 min compared to the recommended time of 25 min; for Session Two, the mean time was 21 min compared to a recommended 26 min.

**Table 4:** Suggested and actual time to deliver each section in Session One and Session Two

Session one			Session two		
Section	Suggested time (min)	Actual time spent delivering intervention mean (*SD*)	Section	Suggested time (min)	Actual time spent delivering intervention (*SD*)
Introduction	1	0:32 (0:22)	Introduction	1	0:21 (0:19)
Assessment of Average Daily Walking	3	1:56 (2:04)	Review of Behaviour Change	8	3:57 (2:29)
What Makes it Easier to Walk? (WMIETW)	4	2:48 (1:46)	Goal re‐evaluation/setting	1	1:09 (1:01)
Walking Experiences task (WE)	5	3:36 (2:07)	Supportive Planning (SP)	8	5:18 (2:59)
Goal Setting	1	1:36 (1:35)	Action Planning (AP)	5	3:19 (2:46)
Action Planning (AP)	8	5:05 (2:36)	Conclusions	3	7:19 (4:48)
Conclusions	3	3:35 (2:47)			
Total	25	19:15 (8:14)	Total	26	21:24 (11:00)

NB: All times are in minutes: seconds.

Providers spent less time than suggested delivering components in which patients were asked to elaborate on their answers to specific activities. In Session One, this was evident during ‘What Makes it Easier to Walk?’ (component 3.2), ‘Walking Experiences’ (component 4.2) and ‘Action Plan’ (component 6.2). In Session Two, this was evident during ‘What Helped Patient to Walk More’ (component 8.4), ‘Supportive Plan’ (component 10.2), and ‘Action Plan’ (component 11.2). During Session Two, conclusions took longer than suggested as the recommended delivery time did not include the time needed to deliver trial procedures at the end of the session.

### Fidelity of delivery according to provider

There was considerable variation between providers regarding the number of intervention components delivered competently across Session One (range 36–93%) and Session Two (range 40–91%). Two providers delivered all sections of the intervention in the order specified, three providers delivered sections in the specified order in 75% of sessions, and three providers delivered approximately half of the sections in the specified order. There was variation between providers for the mean time of delivery of Session One (range 8–19 min) and Session Two (6–17 min). The longest session recorded was 65 min in length; the shortest session was 6 min.

### Treatment fidelity related to receipt

Patients’ receipt of the intervention content differed by type of section and component. Five themes related to treatment receipt were identified: (1) motivational components of the intervention, including those focused on enhancing self‐efficacy; (2) volitional components of the intervention, including components to support effective planning; (3) monitoring; (4) role of the provider; and (5) the extent to which hopes and expectations were realized. The five themes are described in detail below with illustrative quotes presented in Appendix (S5).

#### Motivational components of the intervention

Three patients struggled to recall completing ‘What Makes it Easier to Walk? section (components 3.1, 3.2), in the motivational phase of Session One. This component required patients to identify and discuss situations in which they found it easy to walk previously from a short questionnaire. The remaining patients were unsure what they were being asked to do and unclear as to the purpose of the component (Q1.1).

The component ‘Walking Experiences’ (components 4.1, 4.2), which involved asking patients to rehearse previous instances where they have successfully completed walks or found such walks easy, was recalled by most patients and was generally perceived as positive, enjoyable, and a useful prompt to consider the benefits of walking. The patients who engaged to the greatest extent with ‘Walking Experiences’ task seemed to associate the motivational component with a heightened intention to increase walking (Q1.2).

In Session Two, ‘Review of Behaviour Change’ (components 8.1–8.4) aimed to provide positive feedback to enhance patients’ self‐efficacy concerning their walking and increase their motivation. Most of the female patients commented that they liked and valued being given positive feedback by their provider (Q1.3). Most of the male patients described being motivated by their own awareness that they had attained their goals or by being informed that they had achieved their goal by their provider but were generally less concerned about being praised for their efforts (Q1.4).

#### Volitional components of the intervention

All the patients spoke with enthusiasm and had a strong recollection of ‘Goal Setting’ (components 5.1, 5.2), in which patients chose to set a goal of increasing their daily walking by either 10 or 20 min per day. Patients enjoyed the sense of ownership associated with setting their own goals and almost all described setting realistic goals that they felt they could achieve as it gave them something sensible to aim for (Q2.1).

Five of the six male patients spoke of the similarity of ‘Goal Setting’ to their experience of setting targets and goals at work (Q2.2). Three patients described a sense of positivity from being told by their provider that their baseline walking was higher than others in the study (Q2.3).

All patients recalled completing their ‘Action Plan’ (Session One components 6.1, 6.2; and Sessions Two components 11.1, 11.2), in which they detailed when, where, how, and with whom they were going to achieve their weekly goal. Most patients perceived value in planning the ways in which they could increase their walking. There appeared to be a difference in the patients’ attitudes to planning their walks, linked to their employment status. For the patients who were working, planning was important; particularly concerning how they would find the time to fit their walks in. By contrast, retired patients were not keen on planning their walks a week in advance and wanted more flexibility (Q2.4).

During Session Two, patients were asked to complete a ‘Supportive Plan’ (components 10.1, 10.2) to identify factors that may support their walking and identify ways to bring these factors about. Some patients had difficulty recalling this component and some patients found it difficult to differentiate this from the ‘Action Plan’ (components 11.1, 11.2). Of the patients that did recall the task, the majority found it difficult to complete (Q2.5). However, one patient found that the ‘Supportive Plan’ was valuable; his understanding of the way in which intervention components linked with, or underpinned other, components enhanced his overall engagement with the intervention (Q2.6).

#### Monitoring

Two resources were used during the walking intervention to monitor patients’ walking behaviour; the ‘Walking Diary’ (Session One component 6.3, Session Two component 11.3) for patients to monitor and self‐report their own walking and the pedometer to objectively record patients’ walking. Whilst all patients recalled being asked to complete a ‘Walking Diary’ at the end of Session One, many emphasized the importance of the pedometer as a means of objectively validating their walking and were not aware that the ‘Walking Diary’ was the primary tool intended for the personal monitoring of their walking. As a possible consequence of the emphasis placed on the pedometer, one patient thought the ‘Walking Diary was not necessary (Q3.1).

Other patients described how the ‘Walking Diary’ had heightened their awareness of their walking, enhanced their commitment, and helped in the development of a routine. Several patients likened the ‘Walking Diary’ to similar resources they had used, or were aware of from weight loss/slimming clubs (Q3.2).

#### Role of the provider

The role of the provider in delivering the intervention was one that was perceived positively by the patients (Q4.1). All of the patients commented on the value of the structure of the intervention in terms of providing an opportunity to revisit their provider. For some, this provided a point of accountability; for others, it was seen as a means of support and encouragement (Q4.2). When asked what she felt were the most useful aspects of the intervention, one patient described the importance of support as she started to increase her walking (Q4.3).

Over half of the patients explained how a significant factor encouraging them to get involved was that they considered they had been selected by their practice to participate. This appeared to increase their motivation to participate (Q4.4).

Three patients commented on the difference in approach to delivering the intervention from what they were either used to when attending general practice, or expecting from the intervention. One patient stated that the structured approach to delivery of the intervention differed to the usual approach for both himself and his provider (Q4.5). However, other patients perceived value in the patient‐centred approach. This contrasted with their usual experiences of being ‘managed’ or their expectation that they would be told what to do (Q4.6).

Three patients expected more suggestions as to how they could incorporate walking into their daily routine. It is unclear whether this was because they were used to suggestions as part of their usual consultations in general practice or that they explicitly expected that the intervention would comprise such techniques (Q4.7).

For a small number of patients, the lack of further sessions was a key concern (Q4.8). One patient commented on her past experiences of the importance of ongoing support and encouragement, particularly when initial enthusiasm may be difficult to maintain (Q4.9).

#### The extent to which hopes and expectations were realized

Around half of the patients perceived that increasing their walking was an achievable challenge, or a means of proving to themselves that they could achieve something positive. This was most evident in patients who were retired, and who reported that they had sufficient time to walk, but were looking for a reason or motivation to do so.

Most of the patients perceived walking would result in a range of positive outcomes, which appeared to be important when initially signing up for the study. Walking was seen by a number of patients as a goal in itself. The majority of patients referred to a range of factors that had or they perceived would motivate them to continue with their walking; this included enjoyment of the local environment, walking for a purpose such as walking the dog, walking as a form of social activity. and the financial benefits of walking compared to driving (Q5.1).

Most of the patients had also specifically considered increasing their activity levels for reasons associated with their health and well‐being, although these were commonly longer‐term goals. This included losing weight, to help with management of a long‐term condition, to enhance fitness and for general improved health and well‐being (Q5.2). Two male patients sought more tangible and/or proven outcomes for their efforts (Q5.3, Q5.4). A number of patients referred to the fact that they perceived a greater sense of personal involvement, understanding, and control as the intervention progressed (Q5.5).

Almost all of the patients cited their intention to continue to walk more and four patients aimed to increase their walking in the longer term (Q5.6, Q5.7). It was these patients who appeared to have gained a particularly strong sense of enjoyment or benefit from their walking (Q5.8).

## Discussion

### Statement of principal findings

Overall adherence to essential intervention components was high (78%). Differences in competence of delivery of specific sections, and their associated components, were identified. Specifically, the components ‘What Makes it Easier to Walk?’, ‘Supportive Plan’ and ‘Action Plan’ were most likely to be delivered correctly, whilst ‘Summary of Plans’ and ‘Summary of Session’ were the least likely to be delivered correctly. This suggests that the provision of activities that require substantial engagement from patients, for example, via the completion of component‐specific worksheets, may support the enhancement of fidelity by ensuring patients actively engage with the behaviour change content. On average, providers completed the sessions in a shorter time than that specified in the protocol. Mean delivery time for Sessions One and Two were 5 and 6 min shorter, respectively, than the protocol specified. Considerable variation was found across providers in terms of their mean fidelity scores (range 36–93%), and session duration (range 6–19 min), indicating substantial disparity in provider competency. The qualitative data identified differences in patient understanding of specific intervention components. Greater comprehension of planning components was reported, whilst motivational components were poorly understood by most of the patients. Gender differences in understanding and interpretation of specific intervention components were also apparent with female patients reporting increased motivation as a result of receiving positive reinforcement of efforts, whilst male patients preferred receiving feedback based solely on goal achievement.

### Strengths and weaknesses

The present study has several strengths. Firstly, the study made use of a comprehensive treatment fidelity framework (Bellg *et al*., 2004), to conduct a methodologically rigorous assessment of the implementation of the delivery of a walking intervention by health care professionals to their own patients. As the staff delivering the intervention in this study were not members of the research team and were delivering the intervention as part of their routine work, the findings of the research are likely to be more representative of routine clinical practice, than many findings reported in the research literature (Lambert *et al*., 2017; Walton *et al*., 2017). However, it is possible that the intensity of contact between the research team and providers, as a result of monitoring and interview participation, may have contributed to the level of adherence identified in the present study. It has been reported that recording sessions to assess fidelity of delivery can in itself serve to enhance fidelity of delivery (Tappin *et al*., 2000).

The inclusion of both qualitative and quantitative methods has allowed for the development of a more complete picture of the phenomenon under investigation, therefore enhancing the credibility of the findings (Creswell & Plano‐Clark, 2011; Olsen, 2004). Furthermore, the inclusion of both providers and patients was a strength given the current lack of research exploring treatment fidelity from both perspectives (Walton *et al*., 2017).

Another strength is that the present study used audio‐recordings of intervention sessions to undertake an objective assessment of fidelity of delivery, representing the gold standard approach advocated in the NIH‐BCC Framework (Bellg *et al*., 2004). Over 50% of all recorded sessions were assessed across eight different providers and were evenly distributed across time, enhancing confidence that the sample of sessions coded is representative of all the sessions delivered in the present study (Schlosser, 2002). Despite difficulties in recording the time taken to deliver intervention components, for example, overlap in delivery of intervention components, we were able to objectively examine inter‐provider variation in dose of delivery and thus demonstrate the substantial differences in this across providers, which is a strength of the current study.

We acknowledge a number of limitations. Although our focus was on one fidelity framework, other frameworks (e.g., Carroll *et al*., 2007; Gearing *et al*., 2011) may also be useful for investigating treatment fidelity. In the present study, a dichotomous scale indicating the presence/absence of a component was used instead of a Likert rating scale, which would have instead provided information about the quality of delivery. Therefore, it is possible that this might have led to higher fidelity estimates in the present study than would have been found if quality of delivery had been assessed instead. Furthermore, the researcher who undertook the coding of the walking intervention sessions was involved in the provider training and competency assessments and was therefore not blind to the provider. Whilst this could potentially lead to bias, we have demonstrated good inter‐rater reliability in the current study with 25% of sessions double‐coded by an independent second coder.

It is also possible that the health care providers involved in the current study were particularly interested in health behaviour change as study involvement was voluntary; therefore, participants may not necessarily be representative of health care providers based within the included practices or more generally. Furthermore, all health care providers in the current study were female and White British; therefore, it is highly likely that the sample included were not demographically representative of health care providers within the included practices.

Similarly, it is also possible that the patients participating in the current study, and specifically those who agreed to participate in the follow‐up interview, were also particularly motivated and not representative of all patients in the included practices. This is also the case in terms of the representativeness of the patient sample in terms of their gender and ethnicity. This is despite a purposive sampling strategy being employed to maximize the variability of patients interviewed in terms of gender, age, and presence of health conditions.

A range of techniques were utilized to encourage provider recording of all intervention sessions, that is, text message and email prompts, team visits, and gift vouchers. However, the impact of this was not monitored. Which of these methods, or a combination of these, was most successful at encouraging adherence to fidelity assessment procedures is unclear. Research exploring the effectiveness of methods for enhancing adherence to fidelity measurements is, however, warranted. Finally, we were unable to investigate one aspect of fidelity assessment as recommended by the NIH‐BCC Framework (Bellg *et al*., 2004), that is, treatment enactment, during this pilot study for pragmatic reasons.

### Addition to current evidence

In accordance with the NIH‐BCC framework (Bellg *et al*., 2004), achievement of almost 80% adherence to essential intervention components can be regarded as high fidelity. The present study has demonstrated a substantially greater level of fidelity than that found in a similar study conducted in primary care, in which mean overall adherence was 45% (Hardeman *et al*., 2008). The inclusion of fewer components and less flexibility in the intervention delivered in the present study, which had the purpose of making delivery easier for providers, may have contributed to these differences in findings.

Treatment fidelity enhancement strategies were embedded in the main efficacy trial of the walking intervention based on the findings of the current study. As such, providers in the main trial received the same formal training as those in the pilot trial, with an explicit focus on ensuring understanding of the importance of the motivational components targeted at increasing self‐efficacy, and were also required to demonstrate delivery competence before entering the trial. Further, all providers received a detailed intervention manual, with short summary sheets and pre‐printed intervention materials, detailing the specified intervention components and instructions for delivery. However, a treatment fidelity *assessment* plan was not maintained throughout the main trial due to a desire to avoid inducing behaviour change due to measurement procedures rather than intervention effects (Miles *et al*., 2018). Thus, monitoring of delivery was not as constant which may have resulted in greater drift in provider skill and adherence to the protocol over time. The positive relationship between consistent fidelity monitoring and fidelity outcomes is therefore supported by the non‐significant results of the main efficacy trial of the same walking intervention (Williams *et al*., 2015).

In the present study, we identified patient‐level (e.g., personal preference) and component‐level (perceived value) factors which may alone or in combination impact on treatment delivery and receipt. Providers’ experiences of delivering the walking protocol and their perspective on the factors that influenced their adherence to this was examined in another qualitative study (Taylor, 2012). Whilst it is beyond the scope of the current paper to present this data in any depth, provider‐level (e.g., usual health care delivery style, delivery competency), and organizational (e.g., gap between training and delivery, appointment procedure)‐level factors that can impact on treatment fidelity in this setting have been identified (Taylor, 2012). This supports the findings of previous research which has highlighted the important influence of organizational and contextual factors on the successful implementation of behaviour change interventions (Carroll *et al*., 2007; Chaudoir, Dugan, & Barr, 2013; Durlak & DuPre, 2008; Taylor *et al*., 2011). Future attempts to improve fidelity may benefit from this wider perspective, and it may be time to update the NIH‐BCC framework (Bellg *et al*., 2004) which does not give these factors much consideration.

Our findings indicated that patients understanding of intervention components aimed at improving self‐efficacy was poorer than for volitional planning components. Further research is required to explore if this is a consistent finding across other interventions given that techniques to improve self‐efficacy are a common feature of physical activity interventions. If this is the case, the delivery of self‐efficacy techniques in future interventions may need to be considered, and further training provided to health care professionals on how best to deliver content aimed at improving self‐efficacy. Fidelity checks embedded in earlier stages of intervention development and feasibility testing would also be important to explore patient receipt of self‐efficacy techniques and identify ways to enhance understanding.

### Conclusions

By conducting a rigorous investigation into fidelity of delivery of a PA intervention within primary care, we have demonstrated that it is possible to deliver a behaviour change intervention with near‐optimal fidelity if best practice fidelity recommendations are implemented. We therefore recommend that methods for the enhancement and assessment of treatment fidelity are consistently implemented within trials of health behaviour change interventions, particularly within physical activity intervention research.

## Conflict of interest

All authors declare no conflict of interest.

## Supporting information


**Appendix S1.** TIDieR checklist.

**Appendix S2.** Patient interview schedule.

**Appendix S3.** Treatment fidelity coding frame.

**Appendix S4.** Thematic framework.

**Appendix S5.** Illustrative quotes supporting identified themes: treatment fidelity related to receipt.

## References

[bjhp12392-bib-0001] Ajzen, I. (1991). The theory of planned behavior. Organizational Behaviour and Human Decision Making Processes, 50, 179–211. 10.1016/0749-5978(91)90020-T

[bjhp12392-bib-0002] Bandura, A. (1997). Self‐efficacy: The exercise of control. New York, NY: Freeman.

[bjhp12392-bib-0003] Bellg, A. J. , Borrelli, B. , Resnick, B. , Hecht, J. , Minicucci, D. S. , Ory, M. , … Czajkowski, S. (2004). Enhancing treatment fidelity in health behavior change studies: Best practices and recommendations from the NIH Behavior Change Consortium. Health Psychology, 23, 443 10.1037/0278-6133.23.5.443 15367063

[bjhp12392-bib-0004] Borelli, B. (2011). The assessment, monitoring and enhancement of treatment fidelity in public health clinical trials. Journal of Public Health Dentistry, 71(s1), S52–S63. https://doi.org/10.1111%2Fj.1752-7325.2011.00233.x 10.1111/j.1752-7325.2011.00233.x PMC3074245 21499543

[bjhp12392-bib-0005] Carroll, C. , Patterson, M. , Wood, S. , Booth, A. , Rick, J. , & Balain, S. (2007). A conceptual framework for implementation fidelity. Implementation Science, 2, 1–9. 10.1186/1748-5908-2-40 18053122 PMC2213686

[bjhp12392-bib-0006] Chaudoir, S. R. , Dugan, A. G. , & Barr, C. H. (2013). Measuring factors affecting implementation of health innovations: A systematic review of structural, organizational, provider, patient, and innovation level measures. Implementation Science, 8, 22 10.1186/1748-5908-8-22 23414420 PMC3598720

[bjhp12392-bib-0007] Creswell, J. W. , & Plano‐Clark, V. L. (2011). Designing and conducting mixed methods research (2nd ed.). Thousand Oaks, CA: SAGE Publications Inc..

[bjhp12392-bib-0008] Darker, C. , French, D. , Eves, F. , & Sniehotta, F. (2010). An intervention to promote walking amongst the general population based on an ‘extended’ theory of planned behaviour: A waiting list randomised controlled trial. Psychology & Health, 25, 71–88. 10.1080/08870440902893716 20391208

[bjhp12392-bib-0009] Department of Health (2011). Start active, stay active – A report on physical activity for health from the four home countries. Retrieved from https://www.gov.uk/government/publications/uk-physical-activity-guidelines

[bjhp12392-bib-0010] Duffy, S. S. , Cummins, S. E. , Fellows, J. L. , Harrington, K. F. , Kirby, C. , Rogers, E. , … and the Consortium of Hospitals Advancing Research on Tobacco (CHART) (2015). Fidelity monitoring across the seven studies in the Consortium of Hospitals Advancing Research on Tobacco (CHART). Tobacco Induced Diseases, 13(1), 29 10.1186/s12971-015-0056-5 26336372 PMC4557818

[bjhp12392-bib-0011] Durlak, J. A. , & DuPre, E. P. (2008). Implementation matters: A review of research on the influence of implementation on program outcomes and the factors affecting implementation. American Journal of Community Psychology, 41, 327–350. 10.1007/s10464-008-9165-0 18322790

[bjhp12392-bib-0012] French, D. P. , Stevenson, A. , & Michie, S. (2012). An intervention to increase walking requires both motivational and volitional components: A replication and extension. Psychology Health & Medicine, 17, 127–135. 10.1080/13548506.2011.592843 21745028

[bjhp12392-bib-0013] French, D. P. , Williams, S. L. , Michie, S. , Taylor, C. , Szczepura, A. , Stallard, N. , & Dale, J. (2011). A cluster randomised controlled trial of the efficacy of a brief walking intervention delivered in primary care: Study protocol. BMC Family Practice, 12, 56 10.1186/1471-2296-12-56 21699717 PMC3141510

[bjhp12392-bib-0014] Fuller, E. , Mindell, J. , & Prior, G. (2017). Health Survey for England 2016. Retrieved from https://digital.nhs.uk/catalogue/PUB30169

[bjhp12392-bib-0015] Gearing, R. E. , El‐Bassel, N. , Ghesquiere, A. , Baldwin, S. , Gillies, J. , & Ngeow, E. (2011). Major ingredients of fidelity: A review and scientific guide to improving quality of intervention research implementation. Clinical Psychology Review, 31, 79–88. 10.1016/j.cpr.2010.09.007 21130938

[bjhp12392-bib-0016] Glasgow, R. E. , Lichtenstein, E. , & Marcus, A. C. (2003). Why don't we see more translation of health promotion research to practice? Rethinking the efficacy‐to‐effectiveness transition. American Journal of Public Health, 93, 1261–1267. 10.2105/AJPH.93.8.1261 12893608 PMC1447950

[bjhp12392-bib-0017] Hardeman, W. , Michie, S. , Fanshawe, T. , Prevost, A. T. , Mcloughlin, K. , & Kinmonth, A. L. (2008). Fidelity of delivery of a physical activity intervention: Predictors and consequences. Psychology & Health, 23, 11–24. 10.1080/08870440701615948 25159904

[bjhp12392-bib-0018] Lambert, J. D. , Greaves, C. J. , Farrand, P. , Cross, R. , Haase, A. M. , & Taylor, A. H. (2017). Assessment of fidelity in individual level behaviour change interventions promoting physical activity among adults: A systematic review. BMC Public Health, 17, 765–778. 10.1186/s12889-017-4778-6 28969669 PMC5625828

[bjhp12392-bib-0019] Lee, I.‐M. , Shiroma, E. J. , Lobelo, F. , Puska, P. , Blair, S. N. , Katzmarzyk, P. T. , & for the Lancet Physical Activity Series Working Group (2012). Impact of physical inactivity on the world's major non‐communicable diseases. Lancet, 380, 219–229. https://doi.org/10.1016%2FS0140-6736(12)61031-9 22818936 10.1016/S0140-6736(12)61031-9 PMC3645500

[bjhp12392-bib-0020] Lorencatto, F. , West, R. , Bruguera, C. , & Michie, S. (2014). A method for assessing fidelity of delivery of telephone behavioural support for smoking cessation. Journal of Consulting and Clinical Psychology, 82, 482–491. 10.1037/a0035149 24294836

[bjhp12392-bib-0021] Lorencatto, F. , West, R. , Christopherson, C. , & Michie, S. (2013). Assessing fidelity of delivery of smoking cessation behavioural support in practice. Implementation Science, 8, 40 10.1186/1748-5908-8-40 23557119 PMC3622616

[bjhp12392-bib-0022] McHugh, M. L. (2012). Interrater reliability: The kappa statistic. Biochemia Medica, 22, 276–282. 10.11613/issn.1846-7482 23092060 PMC3900052

[bjhp12392-bib-0023] Michie, S. , Hyder, N. , Walia, A. , & West, R. (2011). Development of a taxonomy of behaviour change techniques used in individual behavioural support for smoking cessation. Addictive Behaviors, 36, 315–319. 10.1016/j.addbeh.2010.11.016 21215528

[bjhp12392-bib-0024] Michie, S. , Richardson, M. , Johnston, M. , Abraham, C. , Francis, J. , Hardeman, W. , Eccles, M. P. , Cane, J. , & Wood, C. E. (2013). The behavior change technique taxonomy (v1) of 93 hierarchically clustered techniques: Building an international consensus for the reporting of behavior change interventions. Annals of Behavioral Medicine, 46(1), 81–95. 10.1007/s12160-013-9486-6 23512568

[bjhp12392-bib-0025] Miles, L. M. , Elbourne, D. , Farmer, A. , Gulliford, M. , Locock, L. , McCambridge, J. , & French, D. P. (2018). Bias due to measurement reactions in trials to improve health (MERIT): Protocol for research to develop MRC guidance. Trials, 19(1), 653 10.1186/s13063-018-3017-5 30477551 PMC6258480

[bjhp12392-bib-0026] Moncher, F. J. , & Prinz, R. J. (1991). Treatment fidelity in outcome studies. Clinical Psychology Review, 11(3), 247–266. 10.1016/0272-7358(91)90103-2

[bjhp12392-bib-0027] Morris, J. N. , & Hardman, A. E. (1997). Walking to health. Sports Medicine, 23, 306–332. 10.2165/00007256-199723050-00004 9181668

[bjhp12392-bib-0028] Murphy, M. H. , Nevill, A. M. , Murtagh, E. M. , & Holder, R. L. (2007). The effect of walking on fitness, fatness and resting blood pressure: A meta‐analysis of randomised, controlled trials. Preventive Medicine, 44, 377–385. 10.1016/j.ypmed.2006.12.008 17275896

[bjhp12392-bib-0029] Murray, J. M. , Brennan, S. F. , French, D. P. , Patterson, C. C. , Kee, F. , & Hunter, R. F. (2017). Effectiveness of physical activity interventions in achieving behaviour change maintenance in young and middle aged adults: A systematic review and meta‐analysis. Social Science and Medicine, 192, 125–133. 10.1016/j.socscimed.2017.09.021 28965003

[bjhp12392-bib-0030] National Institute for Health and Care Excellence (2013). Physical activity: Brief advice for adults in primary care (Public health guideline PH44). Retrieved from https://www.nice.org.uk/guidance/ph44

[bjhp12392-bib-0031] Ogilvie, D. , Foster, C. E. , Rothnie, H. , Cavill, N. , Hamilton, V. , Fitzsimons, C. F. , & Mutrie, N. (2007). Interventions to promote walking: Systematic review. British Medical Journal, 334, 1204 10.1136/bmj.39198.722720.BE 17540909 PMC1889976

[bjhp12392-bib-0032] Olsen, W. (2004). Triangulation in social research: Qualitative and quantitative methods can really be mixed In Holborn M. (Ed.), Developments in sociology (pp. 103–118). Lancashire, UK: Causeway Press.

[bjhp12392-bib-0033] Quested, E. , Ntoumanis, N. , Thøgersen‐Ntoumani, C. , Hagger, M. S. , & Hancox, J. E. (2006). Evaluating quality of implementation in physical activity interventions based on theories of motivation: Current challenges and future directions. International Review of Sport and Exercise Psychology, 10, 252–269. 10.1080/1750984X.2016.1217342

[bjhp12392-bib-0034] Resnick, B. , Bellg, B. , DeFrancesco, C. , Breger, R. , Hecht, J. , Sharp, D. L. , … Czajkowski, S. (2005). Examples of implementation and evaluation of treatment fidelity in the BCC studies: Where we are and where we need to go. Annals of Behavioral Medicine, 29, 46–54. 10.1207/s15324796abm2902s_8 15921489

[bjhp12392-bib-0035] Ritchie, J. , Lewis, J. , & Elam, G. (2003). Designing and selecting samples In Ritchie J. & Lewis J. (Eds.), Qualitative research practice: A guide for social science students and researchers (pp. 79–110). London, UK: SAGE Publications.

[bjhp12392-bib-0036] Ritchie, J. , & Spencer, L. (1994). Qualitative data analysis for applied policy research In Bryman A. & Burgess R. G. (Eds), Analysing qualitative data (pp. 173–194). London, UK: Routledge.

[bjhp12392-bib-0037] Sanchez, A. , Bully, P. , Martinez, C. , & Grandes, G. (2015). Effectiveness of physical activity promotion interventions in primary care: A review of reviews. Preventive Medicine, 76, S56–S67. 10.1016/j.ypmed.2014.09.012 25263343

[bjhp12392-bib-0038] Schlosser, R. (2002). On the importance of being earnest about treatment integrity. Augmentative and Alternative Communication, 18, 36–44. 10.1080/aac.18.1.36.44

[bjhp12392-bib-0039] Sniehotta, F. F. , Schwarzer, R. , Scholz, U. , & Schüz, B. (2005). Action planning and coping planning for long‐term lifestyle change: Theory and assessment. European Journal of Social Psychology, 35, 565–576. 10.1002/(ISSN)1099-0992

[bjhp12392-bib-0040] Tappin, D. M. , Lumsden, M. A. , McIntyre, D. , Mckay, C. , Gilmour, W. H. , Webber, R. , … Currie, F. (2000). A pilot study to establish a randomized trial methodology to test the efficacy of a behavioural intervention. Health Education Research, 15, 491–502. 10.1093/her/15.4.491 11066466

[bjhp12392-bib-0041] Taylor, C. A. (2012). Investigating fidelity of health behaviour change interventions in general practice(Unpublished doctoral dissertation). Coventry University Warwick University, Coventry, United Kingdom. Retrieved from https://curve.coventry.ac.uk/open/items/961d5cd9-b24d-4b0b-8be5-2010a539b7ab/1/

[bjhp12392-bib-0042] Taylor, C. A. , Shaw, R. L. , Dale, J. , & French, D. P. (2011). Enhancing delivery of health behaviour change interventions in primary care: A meta‐synthesis of views and experiences of primary care nurses. Patient Education and Counseling, 85, 315–322. 10.1016/j.pec.2010.10.001 21051174

[bjhp12392-bib-0043] Walton, H. , Spector, A. , Tombor, I. , & Michie, S. (2017). Measures of fidelity of delivery of, and engagement with, complex, face‐to‐face health behaviour change interventions: A systematic review of measure quality. British Journal of Health Psychology, 22, 872–903. 10.1111/bjhp.12260 28762607 PMC5655766

[bjhp12392-bib-0044] Williams, N. H. (2011). Promoting physical activity in primary care. British Medical Journal, 343, d6615 10.1136/bmj.d6615 22058135

[bjhp12392-bib-0045] Williams, S. L. , Michie, S. , Dale, J. , Stallard, N. , & French, D. P. (2015). The effects of a brief intervention to promote walking on theory of planned behavior constructs: A cluster randomized controlled trial in general practice. Patient Education Counseling, 98, 651–659. 10.1016/j.pec.2015.01.010 25677127

[bjhp12392-bib-0046] World Health Organization (2010). Global recommendations on physical activity for health. Retrieved from http://www.who.int/dietphysicalactivity/factsheet_recommendations/en/ 26180873

[bjhp12392-bib-0047] World Health Organization (2017). Physical activity. Retrieved from http://www.who.int/topics/physical_activity/en/

