# Supplementary material for: Translating a walking intervention for health professional delivery within primary care: A mixed‐methods treatment fidelity assessment
Source: Br J Health Psychol. 2019 Nov 19;25(1):17–38. doi: 10.1111/bjhp.12392 (PMC7003875; doi:10.1111/bjhp.12392)
Supplement: Supplementary file 2 — Appendix S2. Patient interview schedule. [file BJHP-25-17-s002.docx]

**Appendix S2: Patient Interview Schedule**

**Introduction and overview**

**General questions**

1. **Before you received the letter about the walking intervention, had you been thinking about doing more physical activity / walking?**

Can you tell me what you thought when you received the letter and information about taking part?

What were your thoughts when you first got the letter about the study?

1. **From the information you received can you describe what you expected from the walking intervention?**

Can you tell me what you thought would be involved in the walking intervention research?

What were your expectations?

What did you think you would have to do?

Can you tell me what you were hoping to get out of taking part?

1. **Now that you have had two walking sessions, can you tell me whether your experience was similar or different to what you were expecting?**

If yes, in what way?

If no, why not? What was different about it? What did you think would be involved?

1. **Thinking back, would you have liked any additional information about the intervention before you first saw the PN/HCA?**

I am now interested in finding out more about what you thought of each of the two sessions: Session 1 then session 2.

**SESSION 1:**

1. **Would you be able to briefly talk me through the first walking session you had with the nurse / HCA? What were the main things that you remember? / What particularly stood out for you?**

What happened in this session? / What activities did you do in the session?

What bits did you find especially helpful / unhelpful?

What bits did you especially like or not like?

1. **Session one components/techniques**

It would now be helpful to consider each of the bits of the walking intervention:

If we go through each part of the intervention, it would be great if you could tell me a little about how you found it?

Assessment of average daily walking - where your nurse/HCA used the pedometer to work out your average daily walking

Did you find this useful / not useful?

What makes it easier to walk? – this was the worksheet with 5 statements about what might make it easier to walk.

What did you think about this activity? / Can you describe how you found doing this activity? / How did you feel about using the worksheet?

Did you find the activity useful / not useful? / What was the main thing you think you got out of doing this activity?

Did you like / dislike the activity?

Walking experiences – this was the worksheet where you thought of a previous occasion where you had walked and identified what made it easy.

What did you think about this activity? / Can you describe how you found doing this activity? / How did you feel about using the worksheet?

Did you find the activity useful / not useful? / What was the main thing you think you got out of doing this activity?

Did you like / dislike the activity?

Goal setting – this was when you set your goal for the next week

How did you find this activity?

Did you select your own goal?

Action planning – when you completed your action plan for the next week

What did you think about this activity? / Can you describe how you found doing this activity? / How did you feel about using the worksheet?

Did you find the activity useful / not useful? / What was the main thing you think you got out of doing this activity?

Did you like / dislike the activity?

**SESSION 2:**

1. **Would you be able to briefly talk me through the second walking session you had with the nurse / HCA? What were the key things that you remember?**

What happened in this session? / What activities did you do in the session?

What particularly stood out for you?

What bits did you find especially helpful / unhelpful?

What bits did you especially like or not like?

1. **Session two components/techniques**

It would now be helpful to consider each of the bits of the walking intervention:

Review of behaviour change + feedback – where the nurse/HCA told worked out your average daily walking on the previous week and gave you feedback)

Did you find this activity useful / not useful?

Goal re-evaluation – when you decided whether to change your goal

Did you find the activity useful / not useful?

Did the activity make sense to you?

Did you like / dislike the activity?

Supportive planning ­ - the worksheet where you considered what you would need to do to meet your goal in the next week

What did you think about this activity? / Can you describe how you found doing this activity? / How did you feel about using the worksheet?

Did you find the activity useful / not useful? / What was the main thing you think you got out of doing this activity?

Did you like / dislike the activity?

Action Planning – where you completed your action plan for the next week

What did you think about this activity? / Can you describe how you found doing this activity? / How did you feel about using the worksheet?

Did you find the activity useful / not useful? / What was the main thing you think you got out of doing this activity?

Did you like / dislike the activity?

Thinking about both the intervention sessions;

1. **Was there anything you particularly liked about the sessions?**

Can you tell me a bit more about that?

1. **Was there anything you particularly disliked?**

Can you tell me a bit more about that?

1. **What bits were the most useful?**
2. **What bits were the least useful?**
3. **What did you think about the resources from the sessions?**

Did you particularly like or dislike any of the resources?

What did you think about the presentation of the resources / how they

looked?

How useful did you find the resources?

We have talked a-bit about the intervention resources; it would be helpful if we could now explore the role of the nurse/HCA in delivering the intervention;

1. **How important do you think the PN / HCA is, in terms of delivering the**

**intervention?**

Was there anything in particular that the PN/HCA did or said that you liked or found helpful? / Anything that was not so helpful?

Do you think the intervention would work as well if you were given the resources to use by yourself?

What do you think helped you most – the nurse /HCA, the intervention, or are both important? In what way?

1. **Can I just double-check; was there any point during the sessions where you felt you weren't clear what you were doing?**

Or *why* you were doing it?

If you were unsure of anything, did the PN/ HCA explain the activity / clarify any bits of it for you?

Did that help to make it clearer?

Did the PN/HCA help you to complete any of the activities? In what way did she help?

1. **Can I ask how you would describe the intervention to a friend or family member in a sentence or two? What do you think the main aims of the intervention are?**
2. **Do you have any further comments to add or questions that you**

**would like to ask about the research?**

**Conclusions and thanks**
