# Supplementary material for: Translating a walking intervention for health professional delivery within primary care: A mixed‐methods treatment fidelity assessment
Source: Br J Health Psychol. 2019 Nov 19;25(1):17–38. doi: 10.1111/bjhp.12392 (PMC7003875; doi:10.1111/bjhp.12392)
Supplement: Supplementary file 3 — Appendix S3. Treatment fidelity coding frame. [file BJHP-25-17-s003.docx]

**Appendix S3- treatment fidelity coding frame**

**Walking Intervention Session One**

**Patient code:**

**Name of coder: Date of coding:**

**Content**

Code 0 for not delivered.

Code 1 for delivered. Code 1 only if technique is definitely present.

Please assign a code: If unsure, make a note on the coding frame and note down the time on the recording for discussion.

If the patient elaborates on an activity on the coding frame without being asked, this scores a 1 as the technique is still present.

**Times of components**

Try to assign exact time that each component was completed (even if component was in wrong order).

In notes box - please try to enter time spent on study/trial procedures i.e. completion of study questionnaires

In notes box please detail interruptions

| **INTERVENTION COMPONENTS / TECHNIQUES:** | | **INCLUDED:**  **Yes =1**  **No = 0** |
| --- | --- | --- |
| **Please note start time of intervention here:** | |  |
|  | **1. INTRODUCTION**  **Score 1 if this is delivered before any other part of intervention** |  |
| **Does provider give an overview of the session?**  Score 1 if overview of session is included | |  |
| **Time on recording following component: (minutes / seconds)** | |  |
|  | **2.ASSESSMENT OF CURRENT AVERAGE DAILY WALKING**  **Score 1 if component immediately follows Introduction** |  |
| **Does provider inform patient of their baseline average daily walking figure (minutes / day)?**  Score 1 if provider states patient's average daily walking | |  |
| **Time on recording following component: (minutes / seconds)**  **i.e. following provider informing patient of av. daily walking and any linked discussion** | |  |
|  | **3. WHAT MAKES IT EASIER TO WALK (WMIETW)**  **Score 1 if component immediately follows assessment of average daily walking** |  |
| **Does provider ask patient to complete worksheet?**  **Score 1 if provider asks patient to complete worksheet and/or explains how to complete** | |  |
| **Does provider elicit reasons for WMIETW from patient?**  Score 1 if provider asks patient to elaborate on their high scoring situations and/or patient elaborates on WMIETW and emphasis is on WMIETW  Score 0 if provider just asks patient to read back the scores for each statement, or if provider reads out high scores but does not ask patient to feedback  Score 0 if the patient does not elaborate on WMIETW | |  |
| **Time on recording following component: (minutes / seconds)** | |  |
|  | **4. WALKING EXPERIENCES**  **(Score 1 if component immediately follows WMIETW)** |  |
| **Does provider ask patient to complete worksheet?**  **Score 1 if provider asks patient to complete worksheet and/or explains how to complete** | |  |
| **Does provider encourage patient to read back their examples / elaborate on their experience further?**  Score 1 if there is discussion between provider and patient before/during/following writing of walking experiences where patient elaborates on their walking experiences  Score 1 if patient is asked to elaborate after completing and/or patient does elaborate  Score 0 if patient completes worksheet but nothing else happens or if provider reads back worksheet and patient does not elaborate | |  |
| **Time on recording following component: (minutes / seconds)** | |  |
|  | **5. GOAL SETTING**  **(Score 1 if component immediately follows Walking Experiences;**  **Score 0 if component follows assessment of average of daily walking)** |  |
| **Does provider offer a goal of 10 or 20 minutes increase in average daily walking?**  Score 1 if a goal of 10 or 20 minutes is offered  Score 1 if a goal that includes both 10 and 20 minutes is offered  Score 0 if any other goal is offered, or no goal is offered | |  |
| **Does patient make final decision on goal?**  Score 1 if the patient makes the final decision (on the goal) (even if there is discussion about the goal before the final decision is made by the patient)  Score 0 if provider suggests / decides on goal | |  |
| **Time on recording following component: (minutes / seconds)** | |  |
|  | **6. ACTION PLANNING / CONCLUSION**  **(Score 1 if component immediately follows Goal setting)** |  |
| **Does provider ask patient to complete action plan?**  **Score 1 if provider asks patient to complete worksheet and/or explains how to complete** | |  |
| **Does provider ask the patient to read back /elaborate on their action plan?**  Score 1 if there is discussion between provider and patient before/during/following writing of action plan (where patient elaborates on their plans for walking) and/or patient is asked to read back/elaborate after completing and/or patient does elaborate/read back after completing  Score 0 if patient completes plan but nothing else happens or if provider reads back action plan  Score 0 if provider prompts patient to help them complete but there is no elaboration from patient | |  |
| **Time on recording following component: (minutes / seconds)** | |  |
| **Does provider explain to the patient how to use the diary?**  Score 1 if provider gives instruction on how to complete diary, and/or encourages patient to do so and/or asks patient to bring completed diary to next session | |  |
| **Does provider ask the patient to summarise what they did in the session?**  Score 1 if provider asks patient to summarise what they did in the session and /or patient summarises session - in which they refer to at least 1 intervention component (i.e. assessment of av. daily walking, WMIETW, walking experiences, goal setting, action planning)  Score 0 if provider summarises session or if patient just says, to increase my walking | |  |
| **Does provider ask the patient to summarise their plans for walking in the next week?**  Score 1 if provider asks patient to summarise plans for walking and/or patient summarises plans for walking (i.e. that patient outlines goal they have set and/or details of action plan and/or refers to completing diary of extra walks they are planning)  Score 0 if provider summarises plans for walking | |  |
| **Does provider end the session in a positive way?**  Score 1 if at least one of following is delivered:   - patient is encouraged to walk - patient is encouraged to display action plan (has to happen during last minute (i.e. end) of session - not just when instructing to complete diary) - patient thanked for coming to session - a general social exchange in which both provider and patient engage and which may enhance rapport - patient praised for their efforts during the session   Score 0 if provider says 'good luck', or 'see you next week' | |  |
| **Time on recording following component: (minutes / seconds)** | |  |
| **Total Score: Components / techniques of intervention:**  **(White boxes - out of 20)**  **YES SCORES: NO SCORES:** | | |
| **Notes on the presence of the following by the provider, i.e.**   - **Encouragement / praise; Positive feedback; Criticism**   **Time spent on; (note start and end time; no need to calculate time)**   - **study/trial procedures i.e. completion of study questionnaires; interruptions**   **Any other notes/comments;** | | |
| **Additional intervention components, i.e.**   - **making suggestions** - **giving Government guidelines on physical activity** - **explaining moderate / brisk walking** | | |

**Walking Intervention Session Two**

**Patient code:**

**Name of coder: Date of coding:**

**Times of components**

Try to assign exact time that each component was completed (even if component was in wrong order).

In notes box - please try to enter time spent on study/trial procedures i.e. completion of study questionnaires

In notes box please detail interruptions

**Content**

Code 0 for not delivered.

Code 1 for delivered. Code 1 only if technique is definitely present.

Please assign a code: If unsure, make a note on the coding frame and note down the time on the recording for discussion.

If the patient elaborates on an activity on the coding frame without being asked, this scores a 1 as the technique is still present.

| **INTERVENTION COMPONENTS / TECHNIQUES:** | | **INCLUDED**  **Yes =1**  **No = 0** |
| --- | --- | --- |
| **Please note start time of intervention here:** | |  |
|  | **1. INTRODUCTION**  **Score 1 if this is delivered before any other part of intervention** |  |
| **Does provider give an overview of the session?**  Score 1 if overview of session is included | |  |
| **Time on recording following component: (minutes / seconds)** | |  |
|  | **2. REVIEW OF BEHAVIOUR CHANGE/FEEDBACK (Score 1 if component immediately follows Introduction** |  |
| **Does provider ask the patient to describe their walking experiences in the last week?**  Score 1 if provider asks patient to describe their walks in last week and/or patient describes their walks, giving details of at least one walk  Score 1 if patient describes their walks without being asked to  Score 1 if provider asks patient' how did you get on?' and patient describes their walks in last week  Score 0 if provider asks patient 'how did you get on?' and patient does not describe their walks in last week | |  |
| **Does provider inform patient of their average daily walking in last week?**  Score 1 if provider states patient's average daily walking  Score 1 if provider states whether patient has or has not met their goal | |  |
| **Does provider give praise / positive feedback for meeting goal/efforts to increase walking?**  Score 1 if provider praises patient for meeting their goal / efforts to increase walking  Score 0 if provider does not praise efforts/achievements or if provider says 'never mind' if the patient has not been able to increase their walking | |  |
| **MET Goal - Does provider ask patient to discuss what in particular helped with their walking (if they met goal)?**  Score 1 if the provider asks and/or the patient describes what has helped them to increase their walking (in this component )  Score 0 if provider does not ask but patient mentions what made it easier, but within another component, i.e. supportive plan  OR  **NOT MET GOAL - If patient has not met goal, and patient describes reasons why they did not met goal, does provider ask patient how they might overcome any barriers/reasons they could not walk (if they did not meet goal)?**  Score 1 the provider asks the patient to describe what might help them overcome barriers (should only happen if patient raises barriers)  Score 1 if patient does not mention barrier and provider does not mention barriers  Score 0 if provider asks about barriers without patient mentioning barriers  Score 0 if patient mentions barriers and provider encourages patient to dwell on these barriers; or does not ask how these might be overcome | |  |
| **Time on recording following component: (minutes / seconds)** | |  |
|  | **3. GOAL RE-EVALUATION / GOAL SETTING**  **Score 1 if component immediately follows review of behaviour change** |  |
| **Does provider offer patient a goal as per the protocol?**  Score 1 if;   - goal was achieved (or almost achieved, i.e. patient increased their walking and was only approx a minute off goal) –provider offers patient to either **maintain** goal or go for **harder goal** (of 5-10 minute increase) - goal was not achieved recommend **maintain** goal or **easier goal** (decrease by 5 minutes)   Score 1 if provider raises issue of goal and patient decides on their goal before provider has chance to offer goal choices (as above) AND provider clarifies how this goal relates to last goal, i.e. 'so you want to maintain?', or checks that the patient feels their goal choice is realistic  Score 0 if any other goal is offered, or no goal is offered | |  |
| **Does patient make final decision on their walking plans (goal) for next week?**  Score 1 if the patient makes the final decision (on the goal) (even if there is discussion about the goal before the final decision is made by the patient)  Score 0 if provider suggests / decides on goal  (The patients’ walking plan / goal can be easier/harder or the same) | |  |
| **Time on recording following component: (minutes / seconds)** | |  |
|  | **4. SUPPORTIVE PLANNING**  **(Score 1 if component immediately follows Goal Re-evaluation / setting)** |  |
| **Does provider ask patient to complete worksheet?**  Score 1 if provider asks patient to complete worksheet and/or explains how to complete | |  |
| **Does provider ask the patient to read back /elaborate on their supportive plan?**  Score 1 if there is discussion between provider and patient before/during/following writing of supportive plan where patient elaborates on their supportive plan and/or patient is asked to read back/elaborate after completing and/or patient does elaborate/read back after completing  Score 0 if patient completes plan but nothing else happens or if provider reads back supportive plan  Score 0 if provider prompts patient to help them complete but there is no elaboration from patient | |  |
| **Time on recording following component: (minutes / seconds)** | |  |
|  | **5. ACTION PLANNING / CONCLUSION**  **(Score 1 if component immediately follows Supportive Plan)** |  |
| **Does provider ask patient to complete action plan?**  Score 1 if provider asks patient to complete worksheet and/or explains how to complete  Score 1 if provider asks patient to consider whether they want to keep the same AP (only if patient met goal and all aspects of their plan worked and they are keeping same goal this week) | |  |
| **Does provider ask the patient to read back /elaborate on their action plan?**  Score 1 if there is discussion between provider and patient before/during/following writing of action plan (where patient elaborates on their plans for walking) and/or patient is asked to read back/elaborate after completing and/or patient does elaborate/read back after completing (this should happen even if patient is keeping same goal and action plan)  Score 0 if patient completes action plan but nothing else happens or if provider reads back action plan  Score 0 if provider prompts patient to help them complete but there is no elaboration from patient | |  |
| **Time on recording following component: (minutes / seconds)** | |  |
| **Does provider ask patient to complete the diary?**  Score 1 if provider gives instruction on how to complete diary, and/or encourages patient to complete one diary each week until their follow-up session and/or asks patient to bring diary to next session and/or advises patient to review action plan before completing a new diary and/or gives patient 2 or more diaries | |  |
| **Does provider ask the patient to summarise what they did in the session?**  Score 1 if provider asks patient to summarise what they did in the session and /or patient summarises session - in which they refer to at least 1 intervention component (i.e. review of walking, goal setting, supportive planning, action planning)  Score 0 if provider summarises session or if patient just says, we've looked at how we can increase my walking | |  |
| **Does provider ask the patient to summarise their plans for walking in the next week?**  Score 1 if provider asks patient to summarise plans for walking and/or patient summarises plans for walking i.e. that patient outlines at least one of,   - the goal they have set - details of their action plan - that they need to record their extra walks on their walking diary   Score 0 if provider summarises plans for walking | |  |
| **Does provider end the session in a positive way?**  Score 1 if at least one of following is delivered:   - patient is encouraged to walk - patient is encouraged to display action plan (has to happen during last two minutes (i.e. end) of session - not just when instructing to complete diary) - patient thanked for coming to session - a general social exchange in which both provider and patient engage and which may enhance rapport - patient praised for their efforts during the session   Score 0 if provider ONLY says, i.e. 'good luck', or 'see you next week' | |  |
| **Time on recording following component: (minutes / seconds)** | |  |
| **Total Score: Components / techniques of intervention: (White boxes - out of 20)**  **YES SCORES: NO SCORES:** | | |
| **Notes on the presence of the following by the provider, i.e.**   - **Encouragement / praise** - **Positive feedback** - **Criticism**   **Time spent on; (note start and end time; no need to calculate time)**  **Time to complete trial questionnaire:**  **Time for photocopying:**  **Time for giving pedometer/instructions for pedometer:**  **Time for booking next appointment:**  **Any other notes/comments;** | | |
| **Additional intervention components i.e.**   - **making suggestions** - **giving Government guidelines on physical activity** - **explaining moderate / brisk walking** | | |
