# Supplementary material for: Translating a walking intervention for health professional delivery within primary care: A mixed‐methods treatment fidelity assessment
Source: Br J Health Psychol. 2019 Nov 19;25(1):17–38. doi: 10.1111/bjhp.12392 (PMC7003875; doi:10.1111/bjhp.12392)
Supplement: Supplementary file 4 — Appendix S4. Thematic framework. [file BJHP-25-17-s004.docx]

**Appendix S4: Thematic Framework:**

**1. Patient engagement - initial and on-going**

1.1 Initial engagement (i.e. specific reason for getting involved, hopes of participation)

1.2 Expectations / Information provision

1.3 Engagement through personal circumstances ( i.e. environment, links with work/retirement, dogs, walking for a purpose, part of routine, weather, age)

1.4 Engagement through support and encouragement from others (i.e. support from family, walking with friends, awareness of others in study)

1.5 Engagement through health outcomes (i.e. focus on health benefits, outcome focussed)

1.6 Patient approach / attitude that may enhance engagement (i.e. accepting ups and downs, not feeling guilty, being realistic, flexibility)

**2: Patient understanding - intervention session 1**

2.1 Assessment of average daily walking / Pedometer

2.2 What makes it easier to walk

2.3 Walking experiences

2.4 Goal setting (refs to stepping stones/small steps)

2.5 Action planning

2.6 Diary

**3: Patient understanding - intervention session 2**

3.1 Assessment of walking / feedback

3.2 Goal re-evaluation

3.3 Supportive plan

3.4 Action planning (and longer term planning skills)

3.5 Diary

**4: Provider and setting of delivery - influence on experience and understanding**

4.1 Role of provider (i.e. providing support, encouragement, positivity)

4.2 Provider making suggestions (i.e. that provider did or patient wanted this)

4.3 Provider as source of longer term support

4.4 Issues concerning general practice context (i.e. invitation coming from general practice, intervention being delivered in general practice)

**5: Positive Patient experience**

5.1 General positive references (i.e. enjoyment, comfortable, value, pleased, personal challenge)

5.2 Heightened self-awareness (i.e. awareness of other lifestyle issue, 'it all adds up', and sense of doing it for self, awareness of intensity of walking)

5.3 Knock-on effects (i.e. additional health benefits, lifestyle changes)

5.4 Walking as a behaviour

**6: Negative patient experience**

6.1 General negative references (i.e. difficult; hard; restricted)

6.2 Wanting / expecting more from provider (i.e. suggestions; encouragement)

6.3 Negative references concerning resources (i.e. repetition of questions; activities; not liking activities; uncertainty of what they should be doing)

6.4 Disappointment (i.e. not achieving goals/expected outcomes)

**7: Direct references regarding understanding (receipt) and delivery**

7.1 Direct references regarding understanding/expectations

7.2 Mis-communication or problems and impact of this i.e. with pedometer

7.3 Direct refs to do with delivery, i.e. feeling on the spot, provider suggestions

7.4 Patient suggestions to improve
