# Supplementary material for: Translating a walking intervention for health professional delivery within primary care: A mixed‐methods treatment fidelity assessment
Source: Br J Health Psychol. 2019 Nov 19;25(1):17–38. doi: 10.1111/bjhp.12392 (PMC7003875; doi:10.1111/bjhp.12392)
Supplement: Supplementary file 5 — Appendix S5. Illustrative quotes supporting identified themes: treatment fidelity related to receipt. [file BJHP-25-17-s005.docx]

**Appendix S5: Illustrative quotes supporting identified themes: Treatment fidelity related to receipt.**

*1. Motivational components of the intervention*

1.1 ‘I found it up here a little awkward, just sort of thinking what are they actually asking me for here? [...] at the end I thought well one or two of these questions are awkward to answer, not because they, they’re not understandable but because they are understandable and I just sort of think why have they asked me this?’ *(Jack, male, 55 and over, hypertension).*

1.2 ‘Actually I felt reasonably motivated to do it and maybe that was because of the questions themselves, although I said I struggled to answer them, it was dawning on me, if you find this all so easy and that, why don’t you do it?’ *(Max, male, 55 and over, heart disease).*

1.3. ‘… was very positive with positive feedback and made me feel good about what I was doing, and I think we all respond to that sort of praise’. Jean *(female, 55 and over, diabetes/asthma)*

1.4 ‘she didn’t give me a lollypop or certificate or anything [...] I didn’t really feel any different’ *(Ajay, male, younger, diabetes).*

*2. Volitional components of the intervention*

2.1. ‘[as it gave] something for you to aim for which is not over the top basically, it’s what you’ve chosen to do’ *(Ruth, female, 55 and over, diabetes).*

2.2. ‘That’s a very good idea because like in a lot of jobs if you’ve got a target, psychologically you’re encouraging yourself to try and achieve it and you don’t want to be disappointed in yourself for failing to achieve it, especially when it is difficult to achieve’ *(James, male, 55 and over, high cholesterol).*

2.3. ‘When she [the provider] told me that people only walked 15 minutes a day I thought crikey at least I’m more than double that. Made me focus and I thought oh hang on why do only 30 minutes a day perhaps I should be doing more and then the more we talked about it the more…I mean I like challenges and 60 minutes a day was a challenge’ *(Martin, male, 55 and over, pre-diabetes).*

2.4. ‘I remember doing that [Action Planning] and if I’m quite honest I found that a little bit restrictive, because I’m retired and really my time is my own’ *(Christine, female, 55 and over, hypertension).*

2.5. ‘I was a bit unsure as to what it was actually meaning, but you know, but also I was just not sure of what to put *anyway’ (Dana, female, younger, hypertension).*

2.6. ‘You look at the sheet blankly at first because you’re thinking well all I’ve got to do is walk a bit more which is very simple but now we’ve got to try and explain it in words but yes yeah it was useful and good [...] it’s helpful because it just identifies in your mind what it is that you're going to try and do and if you’d identified what you're going to try and do, it’s then easier to do it’ *(James, male, 55 and over, high cholesterol)*

*3. Monitoring*

3.1. ‘[walking diary] wasn’t great for me; I didn’t feel it was necessary’. *Max (male, 55 and over, heart disease)*

3.2. ‘I had to write in my walks each day and that is good, I find that really good and years ago when I went to Weight Watchers you had to fill in a diary, a daily diary, every meal time you had to write down what you eat and that sort of thing and I think that is the way to do it, to actually, until you get into a routine’ *(Maggie, female, 55 and over, hypertension).*

*4. Role of the provider*

4.1. ‘I just felt that she [provider] made me feel very positive’ *(Jean, female, 55 and over, diabetes/asthma).*

4.2. ‘I think perhaps you need someone to speak to, to let you know if you're doing it right or wrong and that sort of little bit of encouragement like I have said before, um goes a long way and I think it could be open to all sorts of “oh no I can’t be bothered” if you were just doing it yourself, whereas you know you are seeing someone, that face to face thing makes a lot of difference, for me it did anyway’ *(Christine, female, 55 and over, hypertension).*

4.3. ‘I think it was coming back to somebody and sort of if you like checking in every week’ *(Maggie, female, 55 and over, hypertension).*

4.4. ‘So you know I felt good in that sense that my doctor was most probably looking that it could help me and you know and to actually pick me’ *(Dana, female, younger, hypertension).*

4.5. I think from her [providers] point of view, the point of it was that this was the way it was being done sort of thing, this is the means by which she’s got to deliver this particular program [...] I suspect she wasn’t entirely convinced about it herself’ *(Peter, male, younger, hypertension)*

4.6. [provider] has taken a really, almost a neutral stance, not wanting to swing you one way or another or persuade you one way or another, I guess I’m used to a slightly different sort of way of being umm almost managed if you like, I’m used to being told what to do I suppose [...] So that was a slightly different style, if anything it’s probably totally appropriate because obviously this is me isn’t it, I have got to set the goals, I have got to see it through and I can understand that’*(Max, male, 55 and over, heart disease)*

4.7. While I have been encouraged to perhaps take part with someone else or walk in different areas, there hasn’t been a lot of input and I don’t mean this in a disrespectful way [...] but there hasn’t been a lot of suggestions *(Christine, female, 55 and over, hypertension).*

4.8. It felt almost like the umbilical cord was being cut a bit early...I can imagine if somebody did need some support on this, you know you might be in free-fall a bit soon, I mean there is a, this is all about discipline, umm and if you are reporting back to somebody every week that is a sort of discipline. It’s a bit like Weight Watchers isn’t it? You know, if you know you're going to go and somebody’s going to put you on some scales and measure you, I mean God who needs to pay for Weight Watchers really you could do it yourself really for what they do, but in all fairness the success is the fact that that is the motivator, it gives you the discipline to do it. It just felt that sort of support level was being removed quite quickly, quite sharply really’ *(Max, male, 55 and over, heart disease)*

4.9. Maybe there ought to be [...] a review every three months to see how it’s going, see if you’re maintaining [...] because I can see the positive results, I’m very keen to continue, but who knows what I’ll be like in October when it’s chucking it down with rain, if I haven’t got, if it’s purely up to me’ *(Jean, female, 55 and over, diabetes/asthma).*

*5. The extent to which hopes and expectations were realised*

5.1. ‘I have got a lot of areas available to walk; but you work full-time and you forget that they are there, you know it in the back of your mind, but, and then it sort of brought to the fore of how lovely it is’ *(Christine, female, 55 and over, hypertension)*

5.2. ‘I want to be able to lose some weight; you know that is a big thing with me’ *(Dana, female, younger, hypertension).*

5.3. ‘…saying we’re going to increase your walking time because it’ll be good for your health; yeah well prove it’ *(Ajay, male, younger, diabetes).*

5.4. ‘If I understood what the results were of me doing whatever I had been doing, however much walking I did, then I’d be far more inclined to continue it, even if I didn’t really enjoy it, because you’re kind of making yourself do things that you don’t necessarily want to do, but at the moment it’s because it’s, in inverted commas, ‘good for you’, what I don’t know is how good it is for me’ *(Peter, male, younger, hypertension)*

5.5. ‘…you start to feel part of what you’re doing. I think with something like this ownership is really important [...] Because you knew what was coming it didn’t feel so, and plus I’d met [provider] for the third session so we were getting on as well’ *(Ajay, male, younger, diabetes).*

5.6. ‘I see this as a long-term thing rather than a short-term’ *(Max, male, 55 and over, heart disease)*

5.7 ‘I shall make this part of my life in future’ *(Jack, male, 55 and over, hypertension).*

5.8. ‘It does make you feel more positive and I think that its, it isn’t just a temporary measure that you’re doing; it’s going to be a long-term commitment really’ *(Maggie, female, 55 and over, hypertension)*
